# Analysis of Weak Fault in Hydraulic System Based on Multi-scale Permutation Entropy of Fault-Sensitive Intrinsic Mode Function and Deep Belief Network

**DOI:** 10.3390/e21040425

**Published:** 2019-04-22

**Authors:** Jie Huang, Xinqing Wang, Dong Wang, Zhiwei Wang, Xia Hua

**Affiliations:** 1 Urumqi Campus, Engineering University of PAP, Urumqi 830001, China; 2 College of Field Engineering, Army Engineering University of PLA, Nanjing 210007, China; 3 Second Institute of Engineering Research and Design, Southern Theatre Command, Kunming 650222, China

**Keywords:** hydraulic system, leakage fault, multi-scale permutation entropy, fault-sensitive IMF, deep belief network

## Abstract

With the aim of automatic recognition of weak faults in hydraulic systems, this paper proposes an identification method based on multi-scale permutation entropy feature extraction of fault-sensitive intrinsic mode function (IMF) and deep belief network (DBN). In this method, the leakage fault signal is first decomposed by empirical mode decomposition (EMD), and fault-sensitive IMF components are screened by adopting the correlation analysis method. The multi-scale entropy feature of each screened IMF is then extracted and features closely related to the weak fault information are then obtained. Finally, DBN is used for identification of fault diagnosis. Experimental results prove that this identification method has an ideal recognition effect. It can accurately judge whether there is a leakage fault, determine the degree of severity of the fault, and can diagnose and analyze hydraulic weak faults in general.

## 1. Introduction

Equipment failure is a gradual process. If appropriate preventive and remedial measures are taken in the early stages of failure development, more serious losses and consequences can be avoided [1]. Therefore, it is of great significance to extract and analyze weak faults [2]. The features of a fault are weak in the early stage for a few reasons: (1) the degree of damage of the components and parts is small, and the fault signal is weak; (2) there is a certain distance between the fault source and the sensor, and the acquisition equipment and environment noise influence the collected signal; and (3) the vibration of the equipment’s other parts cover up the fault signal to some extent. Therefore, the features of a weak fault are usually difficult to extract, and it is very important to select an appropriate feature extraction method that can extract the information closely related to the fault (namely fault-sensitive information) [3]. In recent years, scholars have proposed some effective methods for the approximation of weak faults, including time domain feature analysis [4], frequency domain feature analysis [5], time-frequency domain feature analysis [6], entropy feature analysis [7,8], correlation detection [9], decomposition algorithm [10,11], stochastic resonance theory [12,13], enhancement algorithm [14,15], combination of filters bank technique and dynamic cumulative sum (DCS) [16], and intelligent algorithm [17]. The sealing characteristic of the hydraulic system structure results in hydraulic faults being concealed and easily affected by random factors. The mapping relationship between the signal characteristics and the system state is also complex [18,19]. Therefore, choosing a right diagnosis method is very critical. Deep belief network (DBN) is a newly proposed deep learning model [20]. It has a strong autonomous learning and reasoning ability that emphasizes learning the hidden representation and highlights the feature expression of data [21]. DBN is useful in solving problems that traditional machine learning algorithms find it difficult to deal with, such as large-capacity data that are high dimensional, redundant, and have nonlinear expression.

In this paper, leakage faults with three different severities are taken as the research object, and a novel method based on multi-scale permutation entropy of fault-sensitive intrinsic mode function (IMF) and DBN is proposed for the identification and analysis of weak hydraulic faults. Experiments show that this method can effectively detect whether there is fault in a hydraulic system and determine the degree of the fault.

## 2. Analysis Method

**Figure 1:**
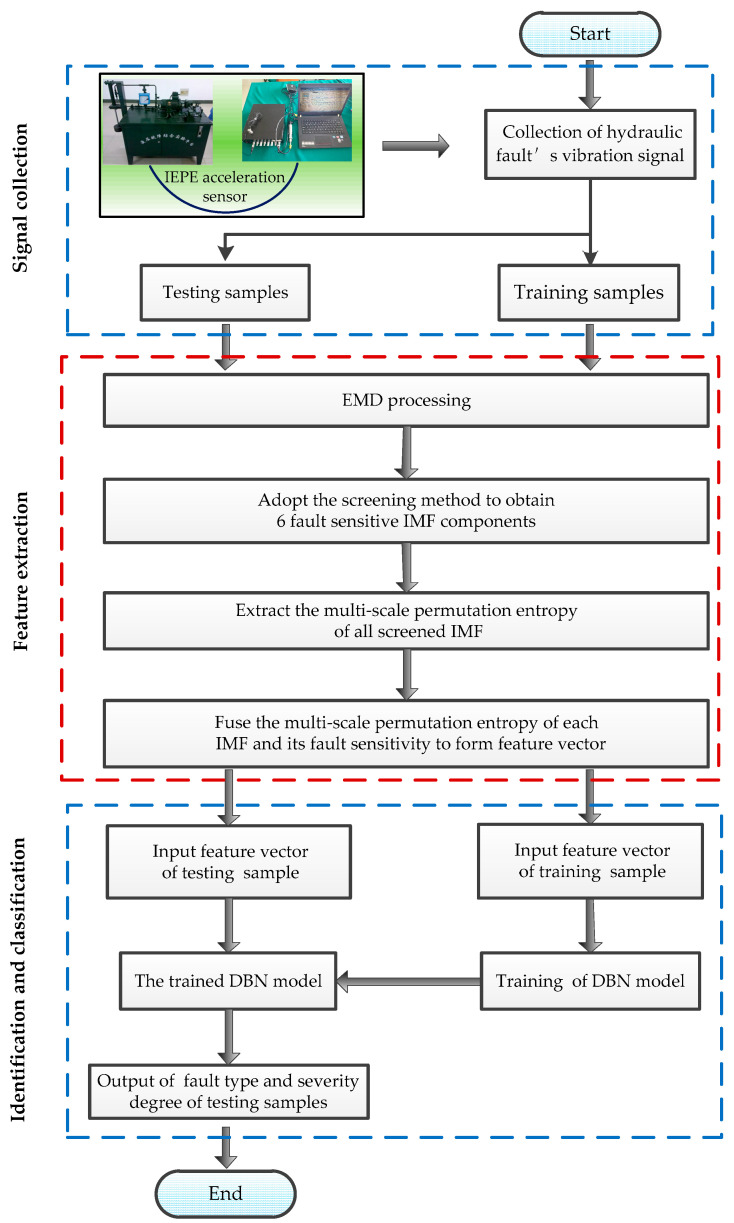
Flow chart of the identification method.

With the aim of detecting hydraulic leakage failure, we sampled the hydraulic vibration signals of a system in normal state as well as systems with slight leakage, moderate leakage, and severe leakage. A novel method is thus put forward for the identification and analysis of weak faults in hydraulic systems. The method combines multi-scale permutation entropy of fault-sensitive IMF and DBN, and its specific steps are as follows (flow diagram is shown in Figure 1):

Step 1: Process the sample signal x(t) by the empirical mode decomposition (EMD) method, which is decomposed into several IMF components and a residue.

Step 2: Adopt the screening method of fault-sensitive IMF components. Get the fault sensitivity $\gamma_{i}$ of each IMF component, eliminate the interference and false IMF components of all the components, and select six effective fault-sensitive IMF components to compose the new IMF groups $\left\{c_{i}(t),i=1,2,\cdots ,6\right\}$.

Step 3: Extract the multi-scale permutation entropy of each $c_{i}(t)$ and merge the fault sensitivity $\gamma_{i}$ with multi-scale permutation entropy of the six IMF components to obtain the feature vector *F* of signal x(t).

(1) $$F=\{\gamma_{1},\gamma_{2},\gamma_{3},\gamma_{4},\gamma_{5},\gamma_{6}{,\mathrm{MPE}}_{1},{\mathrm{MPE}}_{2},{\mathrm{MPE}}_{3},{\mathrm{MPE}}_{4},{\mathrm{MPE}}_{5},{\mathrm{MPE}}_{6}\}$$

Step 4: Repeat Steps 1–3 and extract the feature vectors of all the experimental signals to compose the feature vector set. This will be used as the input of classifier after normalization processing.

Step 5: Divide the feature vector set into the training set and the testing set and establish the most optimal DBN classification model by training and testing its network.

## 3. Multi-Scale Permutation Entropy

Permutation entropy (PE) is a method used to detect the randomness and dynamic mutability of time series as proposed by Bandt and Pompe [22]. It has the advantages of simple calculation, fast processing, strong anti-noise ability, and suitability for online monitoring. On the basis of permutation entropy, Aziz and Arif [23] presented the concept of multi-scale permutation entropy (MPE) to measure the complexity and randomness of time series at different scales.

### 3.1. Permutation Entropy

Permutation entropy is based on the comparison of adjacent data without considering the specific value of data, which can effectively avoid noise interference and reduce the complexity of calculation. The specific calculation principle is as follows:

Step 1: Conduct the reconstruction of phase space for time domain signal sequence x(i) with a length of *N*. The reconstruction parameters’ embedding dimension and time delay are *m* and τ, respectively.

(2) $$\{\begin{array}{c} X(1)=\{x(1),x(1+\tau ),\cdots ,x(1+(m-1)\tau )\}\\ \vdots \\ X(i)=\{x(i),x(i+\tau ),\cdots ,x(i+(m-1)\tau )\}\\ \vdots \\ X(N-(m-1)\tau )=\{x(N-(m-1)\tau ),x(N-(m-2)\tau ),\cdots ,x(N)\} \end{array}$$

Step 2: Sort arbitrary sequence X(i)={x(i),x(i+τ),⋯,x(i+(m−1)τ)} in ascending order, that is

(3) $$X(i)=\{x(i+(j_{1}-1)\tau )\leq x(i+(j_{2}-1)\tau )\leq \cdots \leq x(i+(j_{m}-1)\tau )\}$$

If $x(i+(j_{p}-1)\tau )=x(i+(j_{q}-1)\tau )$ exists, arrange them according to the values of $j_{p}$ and $j_{q}$. For example, when $j_{p}<j_{q}$, then $x(i+(j_{p}-1)\tau )\leq x(i+(j_{q}-1)\tau )$.

Step 3: Calculate the symbol sequence. According to Step 2, a set of symbol sequence S(l) that meets the criteria can be obtained for any sequence X(i).

(4) $$S(l)=[j_{1},j_{2},\cdots ,j_{m}]$$

where l=1,2,⋯,m!

For different symbols $\left[j_{1},j_{2},\cdots ,j_{m}\right]$ of *m*, there are m! different arrangements, that is, m! different symbol sequences.

Step 4: Calculate the permutation entropy. For each symbol sequence, the probability of its occurrence is $P_{l}$, satisfying $\sum_{l=1}^{m!}P_{l}=1$. According to the Shannon entropy, the permutation entropy of time domain signal sequence x(i) is defined as follows:

(5) $$H_{p}(m)=-\sum_{l=1}^{m!}P_{l}\ln P_{l}$$

when $P_{l}=1/m!$, $H_{p}(m)$ gets the maximum value ln(m!), and $H_{p}(m)$ is standardized in turn.

(6) $$H_{p}=H_{p}(m)/\ln(m!)$$

The larger the value of $H_{p}$, the more random is the time domain sequence and the more dispersed is the signal’s energy. The smaller the value of $H_{p}$, the more regular is the signal sequence and the more concentrated is the signal’s energy, meaning the higher the probability of failure. According to experience, the value of *m* is generally 3–7, while the value of delay τ, which has little effect on the signal’s entropy, is advisable to be 1.

### 3.2. Multi-Scale Permutation Entropy

Multi-scale permutation entropy, which is the permutation entropy of time series at different scales, adds a process of coarsening time series on the basis of permutation entropy. The steps are as follows:

Step 1: Roughen the time series x(i) with the scale *s* to obtain the coarsening sequence $\left\{{y_{j}}^{\left(s\right)}\right\}$:

(7) $${y_{j}}^{\left(s\right)}=\frac{1}{s}\sum_{i=(j-1)s+1}^{js}x(i)\begin{array}{cc} & j=1,2,\cdots ,[N/s \end{array}]$$

In the above formula, [N/s] means the integral of N/s; generally, the scale factor is s∈[1,12]. Obviously, when *s =* 1, the coarsening sequence is the original sequence. When *s* > 1, the coarsening sequence length is [N/s].

Step 2: Calculate the permutation entropy of all coarse-grained sequences and obtain the permutation entropy sequence corresponding to the scale factor.

## 4. Structure and Training of DBN

The deep belief network is a probability generation model, which is composed of multiple restricted Boltzmann machines (RBMs). The learning of DBN includes unsupervised training and supervised training. The learning process of deep belief network can be summarized into two parts: (1) the unsupervised layer-by-layer learning process of forward stack RBM from low level to high level and (2) the supervised and fine-tuned learning process from high level to lower level [24].

**Figure 2:**
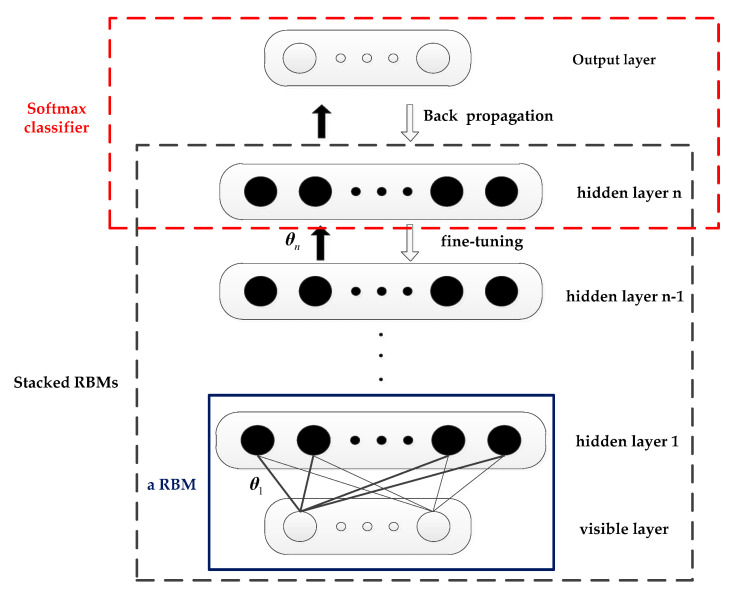
Structural diagram of deep belief network (DBN).

DBN can extract deep features from the complex input data using layer-by-layer greedy learning of multi-layer RBM. A supervised classifier is added on the top-level RBM to form a complete DBN classifier, as shown in Figure 2. The DBN consists of an unsupervised RBM and a supervised Softmax classifier. The visible layer accepts the input data and forms RBM1 with the hidden layer 1, the hidden layers 1 and 2 form the RBM2, and so on to form RBMn. The hidden layer n and the output layer form the Softmax classifier.

DBN’s learning includes unsupervised training and supervised training. Unsupervised layer-by-layer training is the main difference between the DBN model and other models. Unsupervised layer-by-layer learning directly maps data from input to output. When the previous RBM training is completed, the output of the hidden layer is used as the next RBM. For the input of the visible layer, the parameters of each RBM are obtained through layer-by-layer training. Conversely, the visible layer is reconstructed with the hidden layer and the parameters obtained by the training, and the weight of the network is adjusted using the difference between the reconstructed layer and the visible layer. In this way, through layer-by-layer learning of the stacked RBM, the high-level features of the original data are obtained, and the deep model is dissolved into a series of shallow networks. For the supervised part, the data label is brought in the top RBM. The back propagation (BP) algorithm is used to distribute the error to each layer of RBM from top to bottom so as to adjust the DBN network structure parameters, further reduce the training error, and finally improve the classification accuracy of the DBN classifier.

## 5. Characteristic Analysis of Leakage Fault with Different Severities

### 5.1. Comprehensive Experimental Platform of Hydraulic Fault

**Figure 3:**
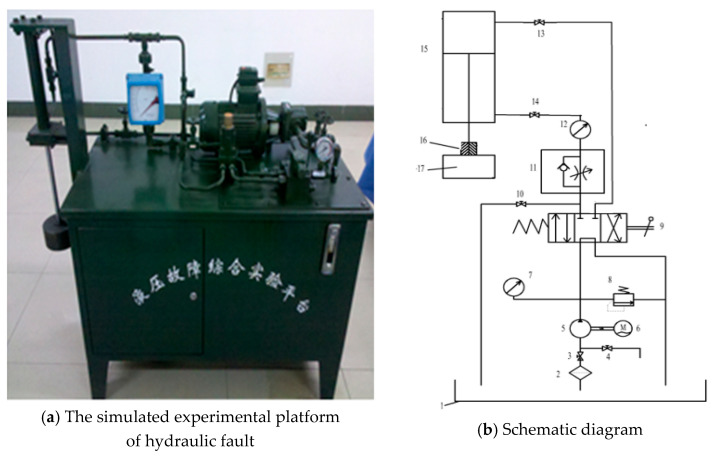
The simulated experimental platform of hydraulic fault (**a**) and its schematic diagram (**b**). 1: fuel tank; 2: suction filter; 3: control valve of oil-absorbing blockage; 4: control valve of cavitation; 5: hydraulic pump; 6: electromotor; 7: piezometer; 8: relief valve; 9: hand-directional valve; 10: control valve of leakage; 11: one-way throttle valve; 12: flowmeter; 13: control valve of oil inlet blockage; 14: control valve of oil outlet blockage; 15: hydraulic cylinder; 16: clamping sleeve; 17: load.

The hydraulic fault signal was measured on the simulated experimental platform of hydraulic fault shown in Figure 3. As can be seen from the schematic diagram of the hydraulic system, typical faults can be simulated in the hydraulic circuit by adjusting the corresponding hydraulic control valve, such as normal working state, leakage, blockage, cavitation, impact, etc. By adjusting the degree of opening or closing of the valve port, fault states of different severities can also be simulated. In addition, multiple valve ports can be combined to form composite failures. In this study, the hydraulic vibration signals of a normal system and systems with slight leakage, moderate leakage, and severe leakage were measured.

### 5.2. Leakage Fault Signal

In this study, the hydraulic state signals of a normal working system and systems with the above three different severities were taken as the research object. The EMD and multi-scale entropy analysis method was used to extract the fault feature, and the DBN was used as the classifier to analyze the weak faults of the hydraulic system. For each state, i.e., normal, slight leakage, moderate leakage, and severe leakage, 100 samples were collected to constitute the experimental sample set. Of these, 70 samples of each state (280 in total) were chosen randomly to form the training set, and the remaining 30 (120 in total) formed the testing set.

**Figure 4:**
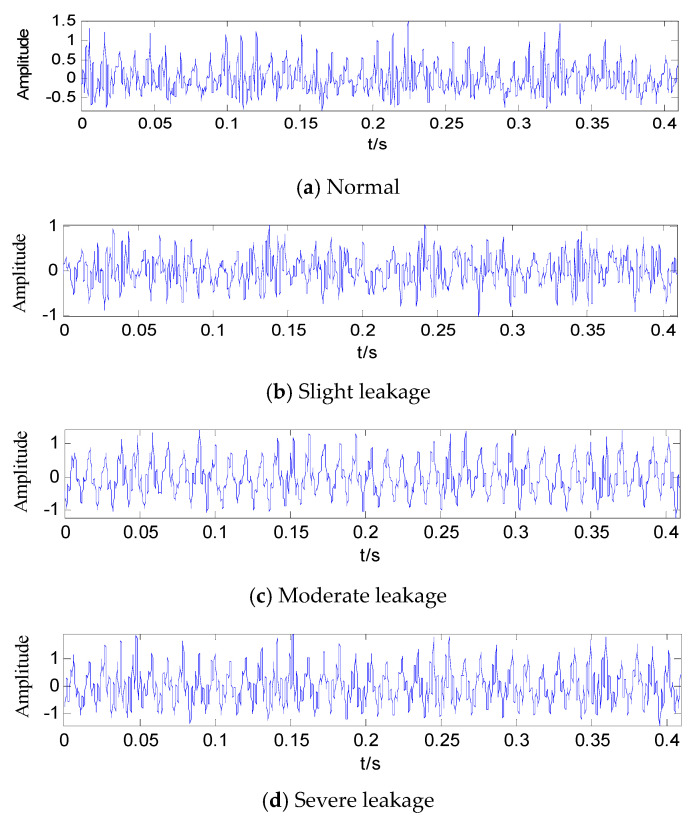
Time domain figures of fault signals with different degrees of leakage. (**a**) Normal state; (**b**) slight leakage; (**c**) moderate leakage; (**d**) severe leakage.

In order to study the characteristic of a weak fault, three kinds of serious leaks were simulated on the hydraulic platform. IEPE acceleration sensor was applied to obtain vibration signals on the hydraulic cylinder. The product type was 122A100, and its range of measured frequency was 0–10 KHz, while the sensitivity was 10.05 mV/ms^2^. During the experiment, considering that the maximum frequency of the collected vibration signals could not exceed 1000 Hz, the sampling frequency was set as 5000 Hz, and the sampling points were set as 2048. The time domain figure of the vibration signals are shown in Figure 4. During the testing, the degree of leakage was controlled by the opening size of the leakage valve in the hydraulic line. As the opening size got bigger, the severity of the leakage increased, the actuator worked more laboriously, and the time taken to complete an upward work was longer. Finally, the actuator stopped working when the opening size reached the limit. When the leakage valve was complete closed, the experimental equipment worked normally, and the uplink time of the actuator was about 1.6 s. When the leakage valve opened a little to simulate a slight leakage fault, the uplink time was about 2 s. When the opening size was doubled to simulate a moderate leakage fault, the uplink time was about 3.5 s. To simulate a severe leakage fault, the leakage valve was opened widely. Here, the actuator was difficult to “crawl”, and the uplink time was about 5 s. From the time domain figures (Figure 4), it is difficult to establish the changes that arose from leakages with different degrees of severities. Therefore, further analysis was deemed necessary.

### 5.3. Spectral Characteristic Analysis of Leakage Fault Signal

**Figure 5:**
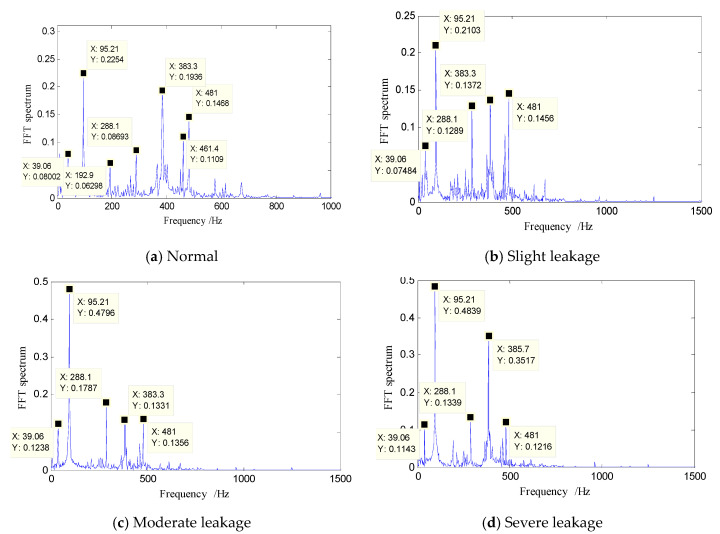
Spectrum figures of fault signals with different degrees of leakage. (**a**) Normal; (**b**) slight leakage; (**c**) moderate leakage; (**d**) severe leakage.

The spectra of normal state, slight leakage, moderate leakage, and severe leakage are shown in Figure 5. It can be seen that, for the same type of fault, even if the degree of severity was different, their frequency components were approximately the same. However, the amplitude of each frequency component varied depending on the degree of severity.

From the perspective of system operation, although the impact of a slight leakage may be small and difficult to visually detect, a weak fault is a turning point in the health status of a system. If measures are not taken in time, the leakage may cause operational deterioration and worsen damage to the equipment. Compared with a normal signal (Figure 5a), when the hydraulic system developed a slight leakage, the frequency components (Figure 5b) were basically unchanged according to frequency analysis. The amplitude of the main frequency component 95.21 Hz (fundamental frequency) changed little, the amplitude of quadruple frequency 383.3 Hz decreased, and the amplitudes of the three-frequency multiplication—288.1 Hz, 383.3 Hz and 481 Hz—were the same.

From a slight leakage to a moderate leakage, the prominent frequency components were still 39.06 Hz, 95.21 Hz, 288.1 Hz, 383.3 Hz, and 481 Hz, but other low-amplitude miscellaneous frequencies were reduced, and the signal’s energy was concentrated on these prominent frequencies. The amplitude of the dominant frequency component 95.21 Hz nearly doubled, and 39.06 Hz and 288.1 Hz increased slightly.

From moderate leakage to severe leakage, the frequency components were still basically unchanged, and the biggest difference was the great increase in frequency component 385.7 Hz (383.3 Hz).

From the above analysis, we can conclude that, when a leak appears in a hydraulic system, as the degree of severity increases, the variations are concentrated in the fundamental frequency 95.21 Hz and its quadruple frequency 383.3 Hz (385.7 Hz). Starting from a slight leakage, as the degree of leakage increases, the amplitude of 95.21 Hz gradually increases. After reaching a certain limit, it stops changing, and the amplitude of 383.3 Hz (385.7 Hz) starts to increase in turn. For other types of faults, a similar rule occurs when their degree of severity increases.

### 5.4. Influence of Parameter Variation on Multi-Scale Permutation Entropy

**Figure 6:**
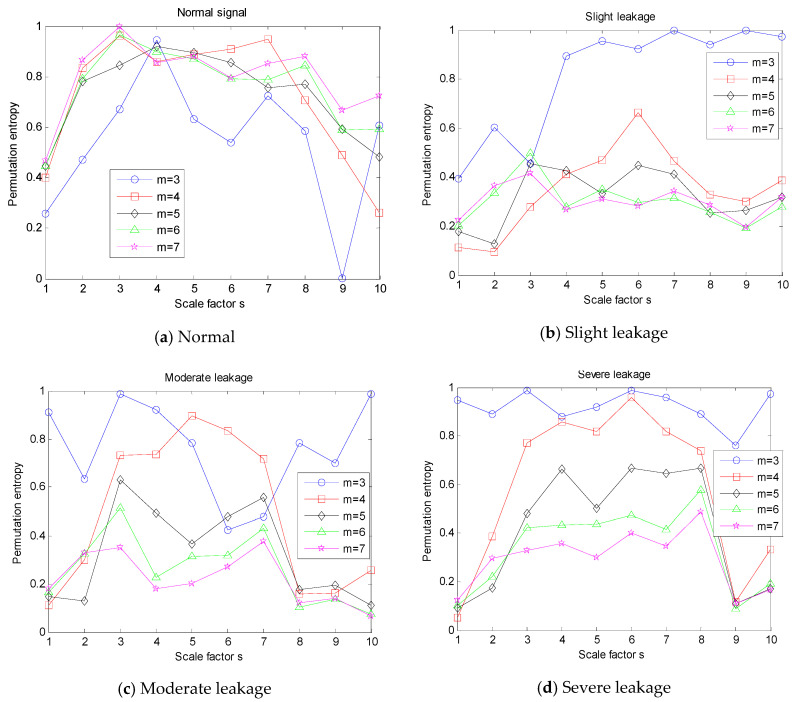
Changes in the multi-scale permutation entropy (MPE) of signals with embedding dimension *m* and scale factor *s*. (**a**) Normal; (**b**) slight leakage; (**c**) moderate leakage; (**d**) severe leakage.

In the analysis of multi-scale permutation entropy, there are two important parameters—The embedded dimension m and scale factor s—That have a great impact on the results. Therefore, before the feature extraction, the influences of m and s need to be discussed [25]. According to previous literature, *m* has the value 3–7 and *s* has the value 1–10. We selected four kinds of typical signals from the experimental sample set to conduct multi-scale entropy analysis, and the results are shown in Figure 6.

According to the multi-scale entropy analysis of signals in Figure 6, the embedding dimension *m* and scale factor *s* have a great impact on the MPE curve, and the difference between the MPE curves of different signals is obvious. Compared with other values, when *m* is 3, the MPE curve has more “outliers”. As the degree of severity deepens, the MPE curves of different m values are more dispersed. When the *m* is fixed, the multi-scale permutation entropy of the signal changes continuously along with scale factor *s*.

## 6. Screening of Fault-Sensitive IMF Components

### 6.1. Calculation of Fault Sensitivity Based on Correlation Analysis

The fault signal is decomposed by EMD to obtain a set of IMF components in which there are some noise interference components and intermediate pseudo components due to the decomposition. As the fault state of the system evolves from a normal state, the fault signal also contains the information component of the normal working state in addition to the fault information. In order to screen out the IMF components closely related to the fault state, except for noise interference components and intermediate pseudo components, the interference effects associated with normal state should also be eliminated. Therefore, a screening method of fault-sensitive IMF components based on correlation analysis is proposed. The specific steps are as follows:

Step 1: Decompose the fault signal x(t) by EMD to obtain the IMF components $\left\{c_{i},i=1,2,\cdot \cdot \cdot ,n\right\}$ and the residual function.

Step 2: Calculate the correlation between the IMF component $c_{i}$ and the original fault signal x(t) and record the correlation coefficient as $\alpha_{i}$.

Step 3: Calculate the correlation between the IMF component $c_{i}$ and the typical normal signal $x_{0}(t)$ and record the correlation coefficient as $\beta_{i}$.

Step 4: Calculate the sensitivity $\gamma_{i}$ of the IMF component $c_{i}$ with the following formula:

(8) $$\gamma_{i}=\alpha_{i}-\beta_{i}$$

Step 5: According to the fault sensitivity $\gamma_{i}$, determine the number of components *k*, select the fault-sensitive IMF components, and rank in descending order according to the size of fault sensitivity, recorded as $\left\{y_{1},y_{2},\cdots ,y_{k}\right\}$. The number of specific fault-sensitive IMF components *k* needs to be weighed in view of the EMD decomposition situation of the overall experimental sample set and the size of each IMF ’s fault sensitivity. In this work, *k* was taken as 6.

### 6.2. Screening Process of Fault Sensitive IMF Components

**Figure 7:**
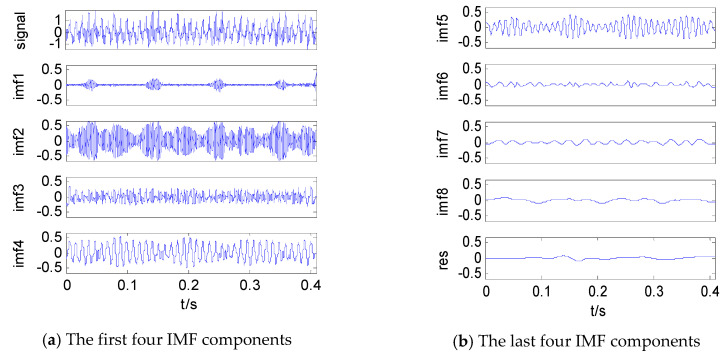
Empirical mode decomposition (EMD) results of a severe leakage fault signal. (**a**) The first four IMF components; (**b**) The last four IMF components.

A severe leakage fault signal was selected in the experimental sample to perform EMD, and the result is shown in Figure 7.

**Figure 8:**
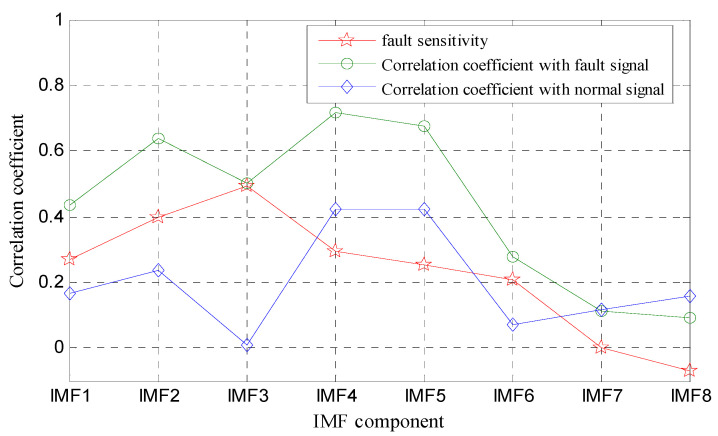
Fault sensitivities of IMF components.

With the decomposition, eight IMF components and a residual function were obtained. According to the above calculation principle of fault sensitivity, we calculated the fault sensitivity of each IMF, as shown in Figure 8. It can be seen from Figure 8 that, among the IMF components, although IMF3′s correlation with the fault signal was only ranked fourth, it was very irrelevant to the normal signals, and its fault sensitivity was the largest. IMF2 had a large correlation with the fault signal, and the correlation with normal signals was not very strong, so its fault sensitivity ranked second. IMF4 and IMF5 were the most relevant to the fault signal, but the correlation with the normal state signal was also large, so their fault sensitivity was not the largest. The fault sensitivities of the first six IMF components were basically greater than 0.2. IMF7′s correlations with the fault signal and normal signal were both similar and small, so its fault sensitivity was close to 0. IMF8′s correlation with the fault signal was less than that with normal signal, so its fault sensitivity was a negative value.

According to the above analysis, the most relevant component to the fault was IMF3, while the second was IMF2. IMF1 and IMF4 were similar, ranking third and four, respectively. The fault sensitivity gradually reduced from IMF3 to IMF8, and the impact was very small after IMF6. Therefore, IMF1–IMF6 were selected as the fault-sensitive IMF components.

## 7. Identification and Analysis of Leakage Faults

In this study, multi-scale permutation entropy based on fault-sensitive IMF was used to conduct feature extraction, and DBN was then combined to identify and analyze weak faults. In this method, fault signal is first decomposed by EMD to obtain IMF components. Second, IMF components sensitive to faults are screened out through correlation analysis, and the fault sensitivity is taken as part of the feature vector. Third, multi-scale permutation entropy feature of the selected fault-sensitive IMF components are extracted, and the obtained multi-scale permutation entropy feature of all IMF components and their corresponding sensitivities are then fused to form the final feature vector. Finally, the DBN is used for identification and analysis.

### 7.1. Feature Extraction Based on Multi-Scale Permutation Entropy of Fault-Sensitive IMF

For the sample set, the fault sensitivity calculation principle was adopted to select the first six IMF components as the fault-sensitive IMF group $\left\{y_{i},i\in [1,6]\right\}$. Multi-scale entropy of each fault-sensitive IMF was extracted under different condition of m=3 and s=10, recording the multi-scale permutation entropy corresponding to IMF component $y_{i}$ as ${\mathrm{MPE}}_{i}$; ${\mathrm{MPE}}_{i}$ is obviously a 10-dimensional vector. Considering the fault sensitivity $\gamma_{i}$ of each component $y_{i}$ at the same time, the feature vector *F* of the fused fault-sensitive IMF components $y_{i}$ was obtained as follows:

(9) $$F=\{\gamma_{1},\gamma_{2},\gamma_{3},\gamma_{4},\gamma_{5},\gamma_{6}{,\mathrm{MPE}}_{1},{\mathrm{MPE}}_{2},{\mathrm{MPE}}_{3},{\mathrm{MPE}}_{4},{\mathrm{MPE}}_{5},{\mathrm{MPE}}_{6}\}$$

It can be seen that the dimension of the feature vector *F* was 66. Using this feature extraction method, the obtained feature vector took into account the correlation between the IMF component and the fault signal itself, strengthened the connection with the fault information, weakened the influence of normal information unrelated to the fault, and utilized the ability of multi-scale entropy analysis method to characterize the characteristics of a weak fault. According to the abovementioned feature extraction method, the feature vector sets of all experimental sample sets were obtained, as shown in Table 1.

### 7.2. DBN Identification of Leakage Fault

**Figure 9:**
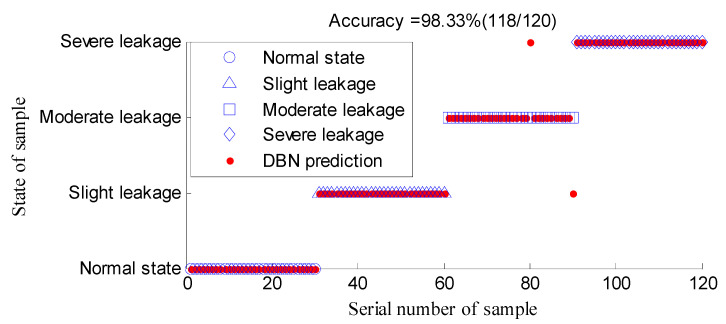
Classification result of DBN.

In this work, a DBN with two hidden layers was selected as the classifier, and the unit number of the hidden layer was 100, that is, the DBN structure was 66-100-100-4, and other structural parameters took the default values. We took 70 group feature vectors of samples in each state (280 in total) in Table 2 to form the training set, and the remaining 30 groups formed the testing set. After normalization, the training and testing sets were used as the input of DBN classifier. By training, the DBN model was obtained, and the testing classification results are shown in Figure 9.

According to the classification results of DBN, 118 of the 120 testing samples were accurately identified, and the classification accuracy reached 98.33%. All the samples of normal state, slight leakage, and severe leakage were accurately recognized. Two moderate leakage samples were misjudged as slight leakage and severe leakage, respectively. The experimental results showed that the identification scheme combining multi-scale entropy feature extraction of fault-sensitive IMF with DBN can effectively detect whether there is a leakage fault and accurately determine the degree of the fault. This effective recognition of weak faults is ideal for meeting the needs of engineering practice.

In order to establish the recognition ability of the proposed method, a comparison of the following was carried out: (1) combining multi-scale permutation entropy of all IMFs with SVM; (2) combining multi-scale permutation entropy of all IMFs with DBN; (3) combining multi-scale permutation entropy of fault-sensitive IMFs with SVM; (4) the proposed approach, i.e., combining multi-scale permutation entropy of fault-sensitive IMFs with DBN. The recognition results are shown in Table 2. It can be seen that the diagnosis results gradually improved from method (1) to (4). Both the feature extraction method based on multi-scale permutation entropy of fault-sensitive IMF and the deep learning method DBN were important in the diagnosis process.

## 8. Conclusions

This study proposes an identification method that combines multi-scale permutation entropy feature extraction of fault-sensitive IMF with DBN for the automatic recognition of weak faults in hydraulic systems. The normal state and three different severities of leakage faults were taken as the object of study, and results showed that this identification method had a good recognition effect. It can effectively detect whether there is a leakage fault and determine the degree of the fault.

The fault signal is first decomposed by EMD, and fault-sensitive IMF screening method is used to select the IMF components. Multi-scale entropy feature of each screened IMF is then extracted to obtain the fault feature information closely related to the weak fault. DBN, a deep learning model adopting the greedy learning algorithm layer-by-layer, is used as a classifier. DBN has a strong ability of autonomous learning and reasoning and is good at dealing with large-capacity, high-dimensional, and nonlinear data. It can well express the complex mapping relationship between the measured signal and the state of the hydraulic equipment state and thus effectively achieve the diagnosis and recognition of hydraulic weak faults. 

## Figures and Tables

**Table 1:** Feature vector of multi-scale permutation entropy based on fault-sensitive IMF components.

Sample Type	Serial Number	Feature Vector																	
		FaultSensitivityγ						Multi-Scale Permutation											
								${\mathrm{MPE}}_{1}$										…	${\mathrm{MPE}}_{6}$
		$\gamma_{1}$	$\gamma_{2}$	$\gamma_{3}$	$\gamma_{4}$	$\gamma_{5}$	$\gamma_{6}$	1	2	3	4	5	6	7	8	9	10		…
**Normal State**	1	0.64	0.18	0.45	0.49	0.96	0.62	0.80	0.99	0.47	0.97	0.95	0.50	0.98	0.90	0.89	0.40		…
	2	0.53	0.18	0.35	0.39	0.67	0.53	0.93	0.99	0.52	0.89	0.83	0.38	0.60	0.82	0.97	0.49		
	⋮					⋮						⋮						…	
	100	0.69	0.55	0.64	0.14	0.27	0.26	0.86	0.95	0.33	0.91	0.65	0.24	0.64	0.42	0.68	0.55		
**Slight Leakage**	1	0.83	0.24	0.17	0.57	0.89	0.53	0.76	0.99	0.63	0.97	0.58	0.31	0.79	0.67	0.66	0.00		
	2	0.47	0.13	0.45	0.35	0.48	0.17	0.93	1.00	0.24	0.92	0.96	0.21	1.00	0.95	0.91	0.69		
	⋮					⋮						⋮						…	⋮
	100	0.48	0.37	0.69	0.32	0.48	0.32	0.96	1.00	0.61	0.95	0.87	0.45	0.78	0.93	0.88	0.64		
**Moderate Leakage**	1	0.43	0.53	0.84	0.50	0.44	0.22	0.80	0.96	0.00	0.33	0.76	0.29	0.79	0.86	0.72	0.61		
	2	0.29	0.10	0.37	0.28	0.25	0.15	0.98	1.00	0.24	0.91	0.48	0.04	0.63	0.95	0.98	0.64		
	⋮					⋮						⋮						…	
	100	0.14	0.17	0.28	0.30	0.36	0.05	0.98	0.97	0.31	0.73	0.88	0.04	0.83	0.84	0.86	0.97		
**Severe Leakage**	1	0.25	0.80	0.41	0.20	0.06	0.07	0.96	1.00	0.43	0.91	0.92	0.14	0.70	0.70	0.99	0.44		
	2	0.43	0.88	0.37	0.13	0.32	0.01	0.77	0.99	0.20	0.98	0.87	0.17	0.78	0.51	1.00	0.68		
	⋮					⋮						⋮						…	
	100	0.26	0.85	0.27	0.28	0.35	0.30	0.15	0.00	0.13	0.00	0.11	0.10	0.38	0.20	0.98	0.71		…

**Table 2:** Comparison of different diagnosis methods.

Number	Feature Extraction	Classifier	Recognition Rate
1	multi-scale permutation entropy of all IMFs	SVM	89.16% (107/120)
2	multi-scale permutation entropy of all IMFs	DBN	90% (108/120)
3	multi-scale permutation entropy of fault-sensitive IMFs	SVM	95.83% (115/120)
4	multi-scale permutation entropy of fault-sensitive IMFs	DBN	98.33% (118/120)
